# Hypoxia in the Blue Mussel *Mytilus chilensis* Induces a Transcriptome Shift Associated with Endoplasmic Reticulum Stress, Metabolism, and Immune Response

**DOI:** 10.3390/genes15060658

**Published:** 2024-05-22

**Authors:** Milton Montúfar-Romero, Valentina Valenzuela-Muñoz, Diego Valenzuela-Miranda, Cristian Gallardo-Escárate

**Affiliations:** 1 Interdisciplinary Center for Aquaculture Research (INCAR), Universidad de Concepción, P.O. Box 160-C, Concepción 4030000, Chile; mmontufar@institutopesca.gob.ec (M.M.-R.); valentina.valenzuela@uss.cl (V.V.-M.); divalenzuela@udec.cl (D.V.-M.); 2 Biotecnology Center, Universidad de Concepción, Concepción 4030000, Chile; 3 Instituto Público de Investigación de Acuicultura y Pesca (IPIAP), Guayaquil 090314, Ecuador; 4 Escuela de Medicina Veterinaria, Facultad de Ciencias de la Naturaleza, Universidad San Sebastián, Concepción 4030000, Chile

**Keywords:** bivalve mollusks, hypoxia, oxidative stress, reoxygenation, gills, transcriptome, metabolism, immunity

## Abstract

The increase in hypoxia events, a result of climate change in coastal and fjord ecosystems, impacts the health and survival of mussels. These organisms deploy physiological and molecular responses as an adaptive mechanism to maintain cellular homeostasis under environmental stress. However, the specific effects of hypoxia on mussels of socioeconomic interest, such as *Mytilus chilensis*, are unknown. Using RNA-seq, we investigated the transcriptomic profiles of the gills, digestive gland, and adductor muscle of *M. chilensis* under hypoxia (10 days at 2 mg L^−1^) and reoxygenation (10 days at 6 mg L^−1^). There were 15,056 differentially expressed transcripts identified in gills, 11,864 in the digestive gland, and 9862 in the adductor muscle. The response varied among tissues, showing chromosomal changes in Chr1, Chr9, and Chr10 during hypoxia. Hypoxia regulated signaling genes in the Toll-like, mTOR, citrate cycle, and apoptosis pathways in gills, indicating metabolic and immunological alterations. These changes suggest that hypoxia induced a metabolic shift in mussels, reducing reliance on aerobic respiration and increasing reliance on anaerobic metabolism. Furthermore, hypoxia appeared to suppress the immune response, potentially increasing disease susceptibility, with negative implications for the mussel culture industry and natural bed populations. This study provides pivotal insights into metabolic and immunological adaptations to hypoxia in *M. chilensis*, offering candidate genes for adaptive traits.

## 1. Introduction

Hypoxia is the dissolved oxygen deficiency in the water column and is a significant stress factor for most marine animals that require oxygen to survive [1,2,3]. It can disrupt biodiversity and productivity in aquatic ecosystems [1,2,3,4]. Recently, the increase in hypoxic areas in coastal systems, primarily caused by the combined action of eutrophication and global warming, has attracted significant attention from the scientific community due to its potential ecological repercussions worldwide [3,5,6,7,8]. In surface waters, dissolved oxygen concentrations result from a balance between oxygen production through photosynthesis, consumption caused by respiration, and exchange with the atmosphere, where the latter tends to maintain dissolved oxygen close to saturation, depending on temperature and salinity [9]. According to projections of increased sea surface temperatures in southern Chile caused by climate change, there would be a decrease in the solubility of oxygen in the water and an intensification of the stratification [10,11,12,13]. Furthermore, climate change affects precipitation and river discharge into fjords [14,15]. This could disturb the thickness and extent of the low-salinity layer at the top of the fjords, slowing down the rate of circulation and renewal of deep waters, thereby affecting bottom oxygen concentrations and resulting in detrimental consequences for fisheries and coastal economies [14,15,16].

Under hypoxia conditions, bivalve mollusks display several physiological and molecular responses as an adaptive coping mechanism for environmental stress [17]. Sessile bivalve mollusks can close their valves or reduce water flow during hypoxic events, decreasing oxygen consumption, energy expenditure, and ATP production [18,19,20]. In contrast, mobile aquatic organisms can migrate away from areas with low oxygen [19,21]. When the duration or severe exposure to hypoxic events exceeds the tolerance of marine organisms, it leads to various detrimental effects, which can be lethal or sublethal, with long-term consequences [5,22]. Hypoxia is involved in molecular mechanisms that trigger mass mortality during the summer, explaining the negative impacts on benthic organisms during these events [23,24]. Adaptation in response to hypoxia is critical for maintaining cellular and organismal homeostasis [25]. Gills play an essential role in gas exchange, where the increase in reactive oxygen species (ROS) caused by hypoxia decreases antioxidant agents, causing cellular damage [26,27,28]. Molecular studies report that the duration of hypoxia stress leads to the cessation of protein synthesis and increased protein catabolism, as well as changes in the urea cycle and the expression of genes associated with apoptosis, inflammatory response mechanisms, and neoplasia [3,18,25,29,30,31]. Hypoxia is a common feature of numerous diseases and a frequent player in several cell malignancies and neoplasia [32]. Under normoxia conditions, the HIF-1α gene is the primary regulator of oxygen homeostasis in bivalve mollusks [33,34], promoting the ability of cells to adapt to hypoxia and playing an essential role in immune system cells [35,36]. Excessive production of reactive oxygen species (ROS) during anaerobic metabolism activates apoptosis or programmed cell death through the intrinsic pathway regulated by the *p53* and *BAX* genes [29,37]. Additionally, metabolic imbalance activates the extrinsic apoptosis pathway through caspases 2 and 3 [38,39]. Increased ROS stimulates the inflammatory pathway by activating the TBK1 gene and the NF-kB transcription factor, which promotes apoptosis [29,40,41,42,43,44,45].

Sequencing technology development based on short and long reads provides an indispensable tool for a better understanding of RNA biology, giving pivotal insights about when and where transcription occurs in response to a set of ecological processes [46,47,48]. Notably, the recently published chromosome-level genome assembly for *M. chilensis* (Hupe, 1854) represents a valuable resource for exploring the molecular responses of mussel’s genomes facing the marine environment [49]. For instance, transcriptome studies conducted in *M. chilensis* generated molecular markers related to environmental and biological stressors [50,51,52]. Furthermore, the exploration of molecular markers linked to immune response resulted in the identification of saxitoxin immunoreceptors present in harmful algal blooms, the use of mitochondrial genes as biomarkers for environmental fluctuations such as temperature and salinity, and the exploration of genes related to shell biomineralization, which functions as protection against predators and anatomical support [50,51,52,53]. These analyses help determine the adaptation of populations when transferred from natural seed banks to aquaculture farms [50,51,52]. These genes could be affected by variations in pH caused by ocean acidification [53].

The Chilean mussel, *M. chilensis* (commonly known as “chorito” in Chile), is Chile’s most commercially crucial filter-feeding bivalve mollusk and holds socio-ecological relevance. Its distribution ranges from the Pacific Ocean coast in central Chile to Patagonia in southern Argentina [54,55,56,57,58] The minimum size for extraction is 5 cm, and individuals can reach up to 8 cm [58]. They are gonochoric, with external fertilization and internal sexual dimorphism [59]. Males have a creamy yellow gonad, while females have an orangish tone [59]. Through their buccal palp, they can sort out food particles, eliminating captured material without ingestion through pseudofeces [60]. They can tolerate a wide range of salinity and are particularly abundant in fjords [55]. 

*M. chilensis* is an essential marine resource because it provides ecosystem services [55,61]. The cultivation of mussels begins with collecting seedlings from their natural environment, which are then transported to farms for growing until they reach commercial size [62]. They are then processed for marketing purposes [62]. The species accounts for 98.4% of the shellfish cultivation in Chile and ranks first in worldwide exports [63,64]. In 2021, 424.3 thousand tons were produced, equivalent to over USD 271 million in exports, with the majority directed to Europe [54,62,63,65]. However, in recent years, the mussel farming industry has faced an increased risk of exposure to hypoxia events mainly caused by upwelling and eutrophication in the Los Lagos Region, where 100% of the seed collection and mussel harvesting occurs [9,63,66]. 

Sessile bivalve mollusks have traditionally been used as indicators of water quality. In this context, the feasibility of using *Mytilus* sp. as an environmental biosensor model organism through the characterization of its transcriptome has been proposed, as it can adapt its metabolism to ecological changes [67,68,69]. In recent decades, hypoxia has caused massive mortality and bivalve mollusks’ stranding along Chile’s central southern coast [22,70,71,72]. Therefore, understanding the tolerance mechanisms of bivalve mollusks to hypoxia is currently of utmost importance in contributing to the sustainability of this industry [3,73,74,75,76,77,78,79,80,81,82,83,84,85]. Thus, the effects of hypoxia on the physiological energetics, intermediary metabolites, cell survival, and inflammatory responses of the genus *Mytilus* suggest that hypoxia significantly affects the adaptation mechanisms of *M. chilensis* [3,29,74]. Despite the number of studies conducted on the subject and the available technology for carrying them out, the molecular mechanisms generated in response to the stress adaptation of mussels caused by hypoxia still need to be discovered.

This study adopted the RNA-seq approach to investigate the transcriptomic profiles of the gills, digestive gland, and adductor muscle of *M. chilensis* under hypoxia and reoxygenation conditions. This work aimed to identify differentially expressed genes and their expression patterns under low oxygen levels to gain a better understanding of transcriptomic regulation in response to hypoxia–reoxygenation stress and to investigate the hypoxia-induced changes in the expression of gene pathways involved in hypoxia regulation in *M. chilensis*. Meanwhile, the differentiated response in each analyzed tissue in *M. chilensis* under experimental hypoxia conditions was investigated. These results provide a deep understanding of the molecular regulatory mechanism in different tissues that adapt to hypoxia–reoxygenation in *M. chilensis*. Additionally, the findings of this study can help develop strategies to mitigate the adverse effects of hypoxia in the mussel farming industry. Therefore, studying the transcriptomic response of the native Chilean blue mussel, *M. chilensis*, to hypoxia is crucial for better understanding marine organism biology and addressing current environmental issues. 

## 2. Materials and Methods

### 2.1. Mussel Acclimation, Hypoxia Challenge, and Sample Preparation

In April 2022, adult mussels measuring 6.26 ± 0.50 cm in length and weighing 18.57 ± 3.85 g were collected from Puerto Montt, Chile. They were transported from the cultivation area to the laboratory, where mussels with shell defects were discarded, and the rest were cleaned to remove shell fouling. The mussels were acclimated for 38 days in a 7-ton fiberglass tank containing water at 12.5 ± 0.94 °C and a 34.5 ± 0.32 ppt salinity. Other water quality parameters were measured during the experiment (pH: 7.2 ± 0.12, dissolved oxygen: 7.5 ± 1.11 mg/L). All mussels were fed a mixture of *Isochrysis* sp. and *Pavlova* sp. once a day before and after the hypoxia challenge. The water was changed daily to remove waste products from the mussels. *M. chilensis* is a native species without risk of extinction or protection status, so no special permits were required for this research. 

**Figure 1:**
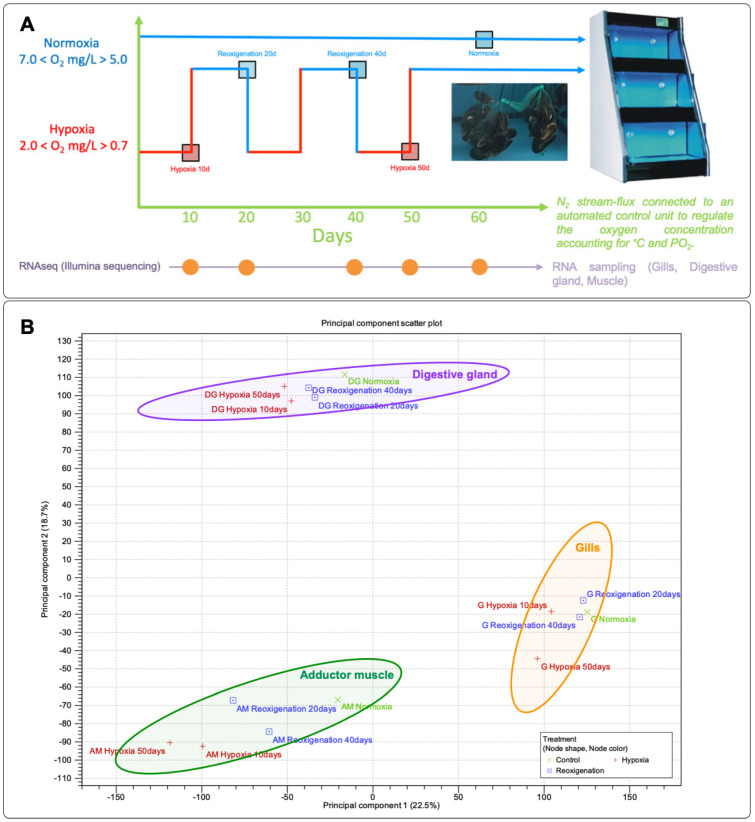
Experimental design and principal component analysis (PCA). (**A**) Experimental design of *M. chilensis* under hypoxia and reoxygenation conditions for 60 days. (**B**) PCA of genes expressed in gills (G), digestive gland (DG), and adductor muscle (AM) under hypoxia and reoxygenation conditions. Circles indicate a differential response by tissue.

The exposure time for the hypoxia assays was based on the average values of the tidal coefficient progression in the area where this organism is cultured. This information is available at the following link: https://tablademareas.com/ (accessed on 7 March 2022). The experimental design for this study is visually outlined in Figure 1A. After acclimation, a total of 480 mussels were randomly divided into two groups and three replicates for the following treatments: (1) control group in normoxia (N1, N2, and N3) maintained at normal oxygen levels (7.2 ± 0.2 mg/L); (2) experimental group subjected to hypoxia (H1, H2, and H3) maintained at a low oxygen concentration (2.0 mg/L). The dissolved oxygen concentration in the recirculation system was controlled daily by injecting nitrogen bubbles until reaching a dissolved oxygen value of 2.0 mg/L. The oxygen content and salinity of the seawater were measured using a multiparameter device (HI9829) (Hanna Instruments Inc., Woonsocket, RI, USA). The water temperature and salinity were 12.5 ± 1 °C and 34.5 ± 0.5 ppt, respectively. For transcriptome sequencing, samples of total RNA were extracted from the gill, digestive gland, and adductor muscle from the control and hypoxia-exposed groups at different time intervals. Each sample was obtained in triplicate. The extracted tissues and extrapallial fluid were frozen at −80 °C for further processing.

### 2.2. RNA Extraction and Library Preparation

Tissues from the gills, digestive gland, and adductor muscle of the control and hypoxia-exposed groups were sampled at 10, 20, 40, 50, and 60 days. The tissues were stored in RNA Later (Ambion, Austin, TX, USA) at −80 °C until RNA extraction for transcriptome sequencing. RNA extractions were performed in triplicate for each experimental group using the Trizol reagent (Invitrogen, Carlsbad, CA, USA) according to the manufacturer’s instructions. Pools made each replicate of 9 individuals. The extracted RNA concentration was measured using the QUBIT 4 instrument (Thermo Fisher Scientific, Pittsburgh, PA, USA), and the quality was determined using the TapeStation 2200 instrument (Agilent Technologies Inc., Santa Clara, CA, USA). Double-stranded cDNA libraries were prepared using the TruSeq RNA Sample Preparation Kit v2 (Illumina^®^, San Diego, CA, USA), following the manufacturer’s instructions, using 1 μg of tissue RNA per group. Each of the three biological replicates per experimental group was sent to the Republic of Korea, where de novo transcriptome sequencing was performed by Macrogen, 180 Inc., using the Illumina platform. The type of read was paired-end, with a read length of 101. 

### 2.3. Transcriptome Analysis and Gene Ontology Annotation

The reads obtained from sequencing were trimmed by quality, and the adapters were removed using the CLC Genomic Workbench software v23 (Qiagen Bioinformatics, Redwood City, CA USA). The trimming parameters included a quality score threshold of 20, a minimum read length of 50 nucleotides, and a sliding window size of 4 nucleotides, and trimming was performed at both ends. Transcriptomic analysis was performed using the *M. chilensis* genome V1 as a reference [49]. RNA-seq analyses were based on all contigs’ normalized TPM (transcripts per million mapped reads) values per sample. The Euclidean distance method was employed to compute the distance metric, and it involved subtracting the mean expression level from 5–6 iterations of k-means clustering. All expression analyses and statistical comparisons included the three biological replicates for each experimental group. The group conducted statistical assessments on TPM values, achieving this by calculating the fold change concerning the control group (normoxia) and then applying Kal’s test for filtering. Transcripts displaying a fold change exceeding |2| and a *p*-value below 0.05 were identified as differentially expressed and subsequently extracted for gene annotation. The *p*-values reported in the analysis were adjusted using the FDR correction to ensure the robustness and reliability of the results. Differentially expressed transcripts were annotated using the BLAST algorithm against the Nr, Nt, egg NOG, Pfam, Swiss-Prot, GO, Ko, KO, and KEGG databases [86,87,88,89,90]. The BLAST E-value cut-off used for annotating the differentially expressed transcripts was set at 1 × 10^−5^. To conduct the analysis of differential gene expression, these transcripts were translated into an Excel matrix. Subsequently, using filters, the transcripts were sorted in descending order, and those showing the highest level of differentiation were selected for generating heatmaps in TBtools Version No.2.007. For the construction of Venn diagrams, online software available at https://www.biotools.fr/misc/venny (accessed on 6 March 2023) was utilized.

Functional enrichment analysis of Gene Ontology (GO) terms was conducted to identify key pathways regulated during the experiment. TBtools, available for free download from https://github.com/CJ-Chen/TBtools-II (accessed on 10 April 2023), was utilized to prepare the gene list for analysis with ShinyGO (http://bioinformatics.sdstate.edu/go/ (accessed on 15 May 2023)), an online gene enrichment tool [91,92]. 

For the TBtools analysis, gene IDs and a corresponding GO annotation file are necessary. The software conveniently provides a downloadable “go-basis.obo” file accessible within its “GO & KEGG” and “GO Enrichment” options.

The analysis focused on the blue mussel, employing a significance threshold (FDR) of 0.05 in ShinyGO. To ensure the clarity of the results, options to remove redundant terms and abbreviate pathways were employed. Visualization plots were generated using the ggplot2 tool implemented in R.

Hierarchical clustering methods were used in the analysis of the heatmaps to classify the data, and Euclidean distance measures were employed to assess the similarity between genes. Not all differentially expressed genes (DEGs) were necessarily included, but rather those considered most relevant for the condition under study. The selection of genes was based on criteria such as their level of differential expression, their association with known biological pathways, and their involvement in physiological functions relevant to the study.

### 2.4. Chromosome Gene Expression (CGE) Analysis 

The unprocessed data from each sequencing process were aligned to the *M. chilensis* genome V1 [49] to evaluate the CGE index, according to Valenzuela-Muñoz et al., 2022 [93]. The CGE index measures the transcriptional variation across the two experimental groups (hypoxia and normoxia). To do this, we determined the average transcript coverage in normoxia within a specific chromosomal region and compared it under different experimental conditions. We applied a threshold range of 2000 to 100,000 reads within a 5-position window to calculate transcript coverage values. The Graph Threshold Areas tool within CLC Genomics Workbench v23 software computed these threshold values for each chromosomal region. Chromosomes exhibiting CGE index values exceeding 60% were visualized alongside the transcript coverage in each dataset using Circo’s software version 0.69-9 [94].

### 2.5. Data Availability

The transcriptome data for mRNA analysis were deposited in the NCBI-SRA (Sequence Read Archive) database under the accession number PRJNA1099139. 

## 3. Results

### 3.1. Principal Component Analysis (PCA) of Gene Expression Profiles in M. chilensis Tissues under Hypoxia and Reoxygenation

In this study, principal component analysis (PCA) was carried out to identify and compare the gene expression patterns in the gills, digestive gland, and muscle, which contributed to 40.5% of the total variability present in the dataset (Figure 1B). The PCA results indicated that PC1 was mainly related to the separation in gene expression observed in the digestive gland tissue. On the other hand, PC2 allowed a clear distinction in gene expression patterns between gill and adductor muscle tissues (Figure 1B). Likewise, along the PC2 axis, a relationship in the expression of transcripts between the gill tissues and the adductor muscle was identified. Generally, it was possible to observe a lower dispersion of the expression data in the digestive gland, gill, and abductor muscle. However, both reoxygenation and hypoxia conditions were observed grouped for all tissues evaluated.

### 3.2. Differential Regulation of Transcripts under Normoxia and Hypoxia Conditions in Multiple Tissues of M. chilensis

**Figure 2:**
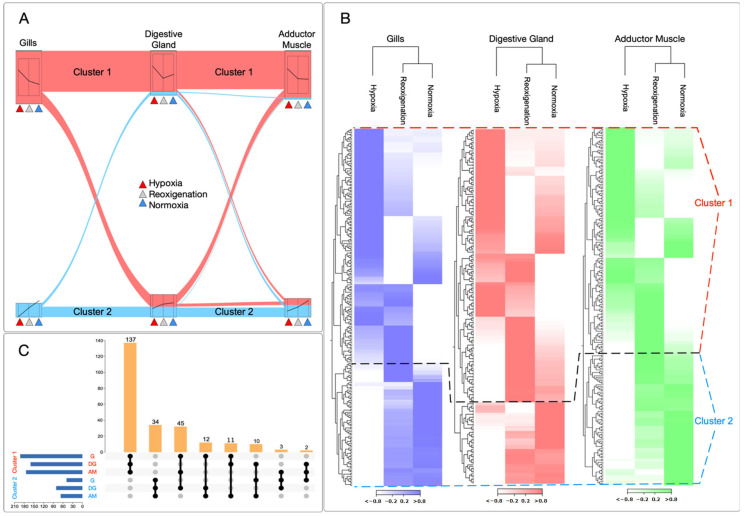
Transcript cluster analysis. (**A**) K-medoids analysis of relevant transcripts for each tissue under normoxia, hypoxia, and reoxygenation conditions. (**B**) Heatmap representation of transcripts for each tissue under normoxia, hypoxia, and reoxygenation conditions. Main clusters 1 and 2 are identified in red and blue, respectively. (**C**) The UpSet plots of transcripts differentially expressed in cluster 1 and cluster 2. Each horizontal bar represents the size of the set of differentially expressed transcripts at a particular time point and treatment. The vertical bars indicate the number of transcripts present in the clusters for each tissue.

To determine gene expression patterns for normoxia, reoxygenation, and hypoxia conditions simultaneously, this study identified two data clusters with marked differences in transcript regulation under normoxia and hypoxia conditions (Figure 2A). Performing RNA-seq analyses revealed distinctive patterns between the experimental groups. For example, cluster 1 showed upregulation of genes in response to hypoxia. In contrast, cluster 2 stood out for the downregulation of genes in all tissues analyzed, including gills, digestive gland, and adductor muscle (Figure 2B). When the UpSet plot was examined to compare the transcriptional regulation of tissues under hypoxic conditions, it was observed that a more significant number of genes were upregulated in cluster 1 in all tissues analyzed. In particular, the gills presented the highest number of upregulated transcripts, followed in order by the adductor muscle and the digestive gland (Figure 2C). In cluster 1, 137 transcripts were identified that were differentially expressed in the tissues evaluated. In cluster 2, 34 transcripts were shared among tissues (Figure 2C).

**Figure 3:**
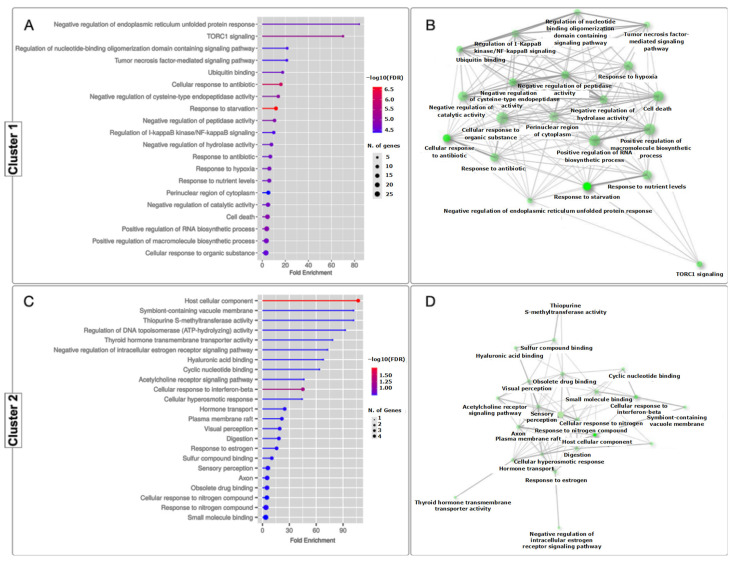
Gene Ontology (GO) enrichment analysis of differentially expressed genes (DEGs). (**A**) GO enrichment analysis of DEGs found in Cluster 1. (**B**) Network analysis of relevant GO terms identified in cluster 1. (**C**) GO enrichment analysis of DEGs found in cluster 2. (**D**) Network analysis of relevant GO terms identifies in cluster 2.

For cluster 1, 20 significantly enriched Gene Ontology (GO) terms were identified (Figure 3A), and for cluster 2, 23 terms significantly enriched GO terms were identified (Figure 3C). These terms formed a network connecting the differentially expressed genes (Figure 3B,D). In total, 43 GO terms were identified in both categories. GO category mapping for both clusters ultimately revealed a wide variety of biological processes, including terms such as “Negative regulation of endoplasmic reticulum unfolded protein response”, “TORC1 signaling”, and “Regulation of nucleotide-binding oligomerization domain containing signaling pathway” for cluster 1 and “Host cellular component”, “Symbiont-containing vacuole membrane”, and “Thiopurine S-methyltransferase activity” for cluster 2. 

**Figure 4:**
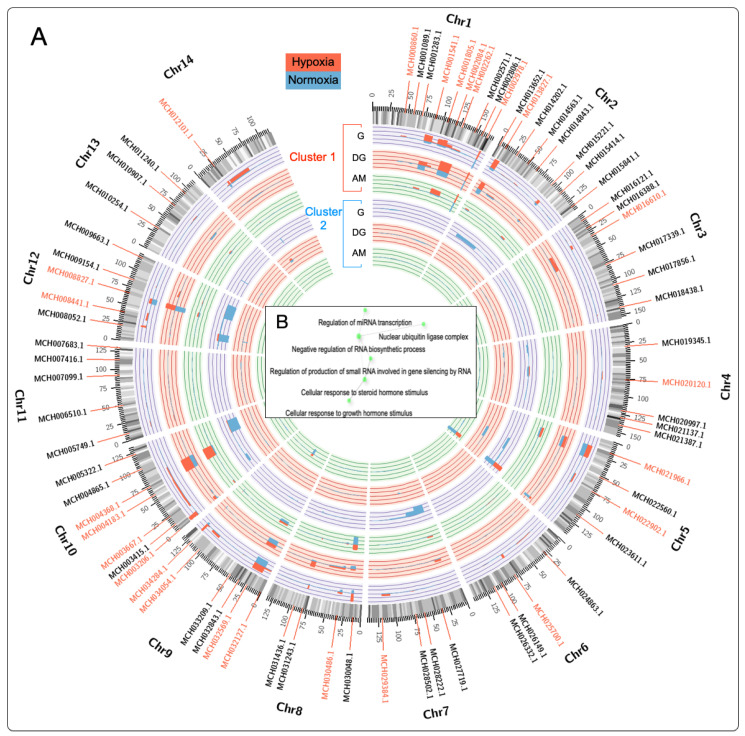
Differentially expressed genes (DEGs) analyzed in *M. chilensis* under hypoxia and normoxia conditions and evaluated through transcriptome analysis in clusters. (**A**) The Circos plot depicts the genomic features of the 14 chromosomes. The DEGs identified in the two analyzed clusters are shown in the Circos plot. From outer to inner circle: gene density, DEG cluster 1, and DEG cluster 2. Red genes represent DEGs associated with hypoxia. Histograms display transcriptional expression levels of G (gill), DG (digestive gland), and AM (adductor muscle). Cluster 1 corresponds to hypoxia (red), and cluster 2 to normoxia (blue). (**B**) Gene Ontology (GO) enrichment network of highly regulated transcripts.

Gene expression cluster analysis was used to identify differentially expressed genes (DEGs) associated with hypoxia in the gill, digestive gland, and adductor muscle tissues (see genes marked in red in Figure 4). Evaluation of the DEGs was performed by analyzing the transcriptome in clusters, visualized using a Circos plot to identify specific loci where the DEGs were highly transcribed. The calculated fold-change values revealed elevated levels of transcription on chromosomes Chr1, Chr9, and Chr10, which exhibit more excellent modulation in response to the hypoxia event (Figure 4A). On the other hand, genes present on chromosomes 5, 7, and 12 showed expression during normoxia events.

The analysis of the *M. chilensis* genome allowed the identification of 25 genes with a high identity index with hypoxia. Among them, the MCH002084.1 gene (haloacid dehalogenase-like hydrolase) located on chromosome 1 stands out, and its association with hypoxia in bivalve mollusks is reported for the first time. The HAD (haloacid dehalogenase-like hydrolase) gene superfamily was activated in response to phosphate deprivation induced by environmental stressors [95,96]. This superfamily comprises a diversity of proteins involved in the hydrolysis of specific substrates, including phosphatases and ATPases [97]. This finding suggests a possible explanation for how oxygen availability may influence the ability of cells to synthesize ATP through aerobic respiration, which could eventually result in metabolic changes and energy production.

When analyzing the genes that were directly involved in the hypoxia events, their relationship with key biological processes such as “Regulation of miRNA transcription”, “nuclear ubiquitin ligase complex”, and “negative regulation of RNA biosynthetic process” was observed (Figure 4B).

### 3.3. Differential Expression Analysis of Transcripts Expressed in M. chilensis Gills under Hypoxic and Reoxygenation Conditions

**Figure 5:**
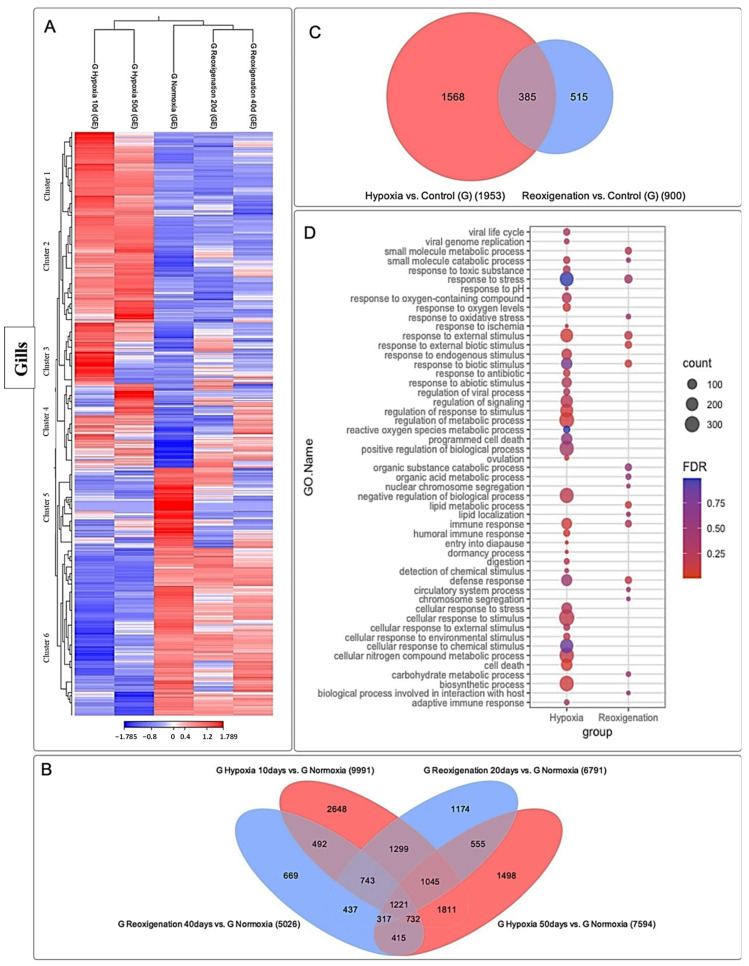
RNA-seq transcriptome analysis in gills illustrating differential RNA-seq expression data. (**A**) The heatmap shows comparisons for each group (normoxia, hypoxia 10 days, reoxygenation 20 days, hypoxia 50 days, and reoxygenation 40 days). Red, positive log fold-change (log FC) indicates higher expression in each treatment; blue, negative log FC. Log FC was calculated using the base 2 logarithm. Grouping was applied by columns (groups compared) and rows (transcripts analyzed), divided into clusters. (**B**) Venn diagram shows the number of unique and overlapping transcripts differentially expressed after exposure to hypoxia–reoxygenation. Sampling was performed at 10 and 50 days of hypoxia and 20 days and 40 days of reoxygenation. A total of 2648 transcripts were differentially expressed in the gill only at 10 days subjected to hypoxia and were not differentially expressed in any other sampling. The most common overlapping transcripts were 1299 differentially expressed at 10 days subjected to hypoxia and 20 days subjected to reoxygenation. Genes regulated in hypoxia are highlighted in red, while those regulated in reoxygenation are highlighted in blue. (**C**) Venn diagram shows overlapping genes differentially expressed compared with aerated controls in hypoxia and reoxygenation in gills. Genes regulated in hypoxia are highlighted in red, while those regulated in reoxygenation are highlighted in blue. (**D**) Function annotation and Gene Ontology (GO) term enrichment analysis of DEGs in upregulated and downregulated genes in response to hypoxia in gills. FDR: false discovery rate. The most representative and significant molecular functions, biological processes, and cellular components are shown. The circumference size indicates the number of DEGs associated with the process, and the dot color indicates the significance of the enrichment (FDR-corrected *p*-values).

The gills subjected to 10 and 50 days of hypoxia experienced significant modifications in their transcriptome, as evidenced in the heatmap (Figure 5A). However, at 20 and 40 days of reoxygenation, the modification in the transcriptome was less pronounced compared to hypoxia (Figure 5A). However, during reoxygenation, recovery and adaptation processes were observed in the gills. Six transcript clusters with different expression patterns were identified (Figure 5A). In particular, clusters 5 and 6 showed upregulation in the control group, whereas clusters 1 to 4 displayed downregulation (Figure 5A). A total of 15,056 transcripts were identified, showing regulation during the 10- and 50-day hypoxia periods, as well as during the 20- and 40-day reoxygenation periods, and a core set of 1221 transcripts were found to be commonly present in all periods of hypoxia and reoxygenation (Figure 5B). The highest amount of differentially expressed transcripts was observed after 10 days of hypoxia, followed by hypoxia at 50 days, reoxygenation at 20 days, and finally reoxygenation at 40 days (Figure 5B). This suggests a more pronounced transcriptome response to hypoxia compared to reoxygenation (Figure 5B). Furthermore, an apparent adaptation of the transcriptome over time in response to hypoxia and reoxygenation exposures was evident (Figure 5B). In the Venn diagram analysis (Figure 5C) it was interesting to observe that the number of differentially expressed transcripts was higher under hypoxic conditions compared to reoxygenation. In particular, the gill presented more transcripts that were only expressed in hypoxia than those that were only expressed in reoxygenation. Transcripts regulated in both hypoxia and reoxygenation were found to be less numerous compared to those expressed exclusively in one or the other state (Figure 5C).

A GO enrichment analysis was performed to examine the response at the metabolic level and in the immune system under hypoxic and reoxygenating conditions in the gills of *M. chilensis* (Figure 5D). Compared to reoxygenation, hypoxia showed an association with a series of significant biological processes and molecular functions (Figure 5D). In hypoxia, GO terms related to stress response, response to external stimuli, regulation of response to stimuli, cellular response to stimuli, and response to oxygen-containing compounds were observed (Figure 5D). In addition, terms related to biosynthetic processes, defensive response, immunological response, and regulation of metabolic processes were highlighted. These results indicate a metabolic and immune system response under hypoxic conditions in the gills of *M. chilensis* (Figure 5D). In the case of reoxygenation, a lower abundance of annotated terms was recorded compared to hypoxia (Figure 5D). However, important terms were still identified, such as small-molecule metabolic processes, stress response, response to external stimuli, lipid metabolic processes, immune response, defensive response, carbohydrate metabolic processes, and biological processes related to interaction with the host (Figure 5D). In summary, the results suggest a marked response at the metabolic level and in the immune system under conditions of hypoxia and reoxygenation in *M. chilensis*, being more pronounced in hypoxia (Figure 5D).

### 3.4. Differential Expression Analysis of Transcripts Observed in the Digestive Gland of M. chilensis under Hypoxic and Reoxygenation Conditions

**Figure 6:**
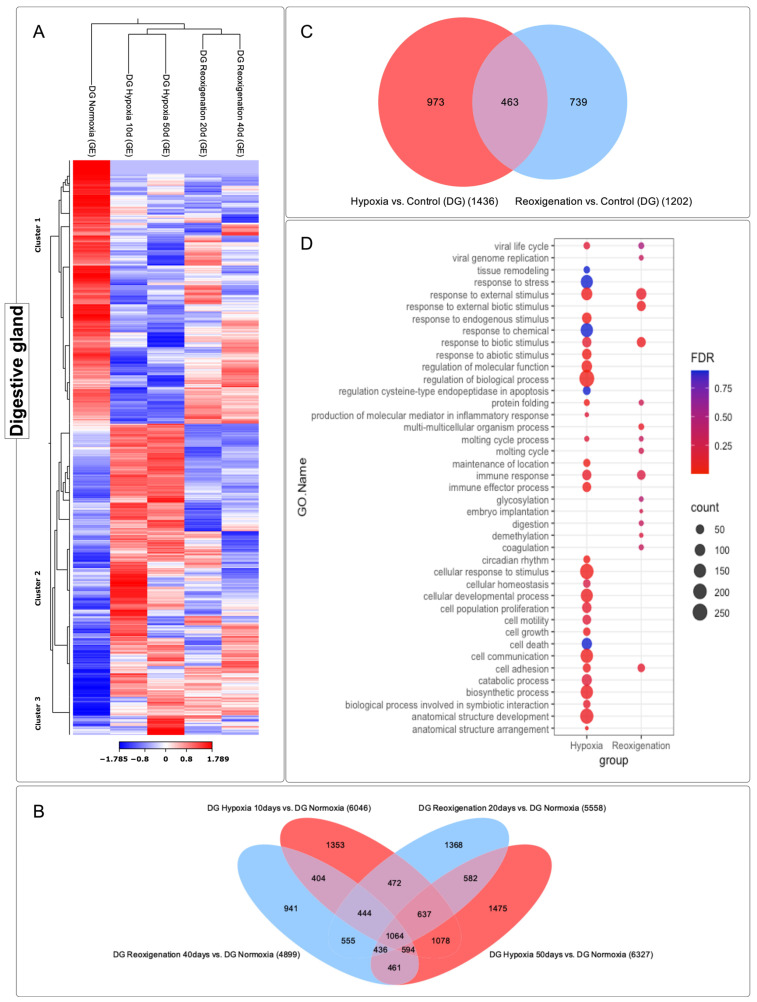
Comparative analysis of the transcriptome in the digestive gland of *M. chilensis*. (**A**) The heatmap shows comparisons for each group (normoxia, hypoxia 10 days, reoxygenation 20 days, hypoxia 50 days, and reoxygenation 40 days). Red, positive log fold-change (log FC) indicates higher expression in each treatment; blue, negative log FC. Grouping was applied by columns (groups compared) and rows (transcripts analyzed), divided into clusters. (**B**) Venn diagram shows the number of unique and overlapping transcripts differentially expressed after exposure to hypoxia–reoxygenation. Sampling was performed at 10 and 50 days of hypoxia and 20 days and 40 days of reoxygenation. A total of 1475 transcripts were differentially expressed in the digestive gland at 50 days subjected to hypoxia and were not differentially expressed in any other sampling. There were 1078 most common overlapping transcripts, which were differentially expressed both at 10 days and 50 days under hypoxia conditions. Genes regulated in hypoxia are highlighted in red, while those regulated in reoxygenation are highlighted in blue. (**C**) Venn diagram shows overlaps of genes differentially expressed compared with aerated controls in hypoxia and reoxygenation in the digestive gland. Genes regulated in hypoxia are highlighted in red, while those regulated in reoxygenation are highlighted in blue. (**D**) Function annotation and Gene Ontology (GO) term enrichment analysis of DEGs in upregulated and downregulated genes in response to hypoxia in the digestive gland. FDR: false discovery rate. The most representative and significant molecular functions, biological processes, and cellular components are represented. The circumference size indicates the number of DEGs associated with the process, and the dot color indicates the significance of the enrichment (FDR-corrected *p*-values).

The digestive gland subjected to 10 and 50 days of hypoxia experienced significant modifications in its transcriptome, as reflected in the heatmap (Figure 6A). However, at 20 and 40 days of reoxygenation, the modification in the transcriptome was less marked compared to hypoxia (Figure 6A). During reoxygenation, a recovery and adaptation process was evident in the digestive gland. Three transcript clusters with different expression patterns were identified in this organ (Figure 6A). Specifically, clusters 2 and 3 showed upregulation. In contrast, cluster 1 showed downregulation (Figure 6A). A total of 11,864 transcripts were identified that underwent regulation during both the 10- and 50-day hypoxia periods and the 20- and 40-day reoxygenation periods, and a core group of 1064 transcripts was found to be consistently present in all the periods of hypoxia and reoxygenation (Figure 6B). The highest number of differentially expressed transcripts was observed at 50 days of hypoxia, followed by hypoxia at 10 days, reoxygenation at 20 days, and finally reoxygenation at 40 days (Figure 6B). These findings suggest a more pronounced response of the transcriptome to hypoxia compared to reoxygenation (Figure 6B). Venn diagram analysis (Figure 6C) revealed that more transcripts were exclusively expressed in response to hypoxia compared to those that underwent regulation during reoxygenation. On the other hand, the transcripts that showed regulation in both the hypoxia and reoxygenation periods turned out to be significantly less numerous compared to those that expressed exclusively in one or the other state (Figure 6C).

### 3.5. GO Enrichment Analysis in the Digestive Gland of M. chilensis under Hypoxia and Reoxygenation Conditions

Hypoxia showed a significant association with a variety of biological processes (Figure 6D). Among the GO terms identified were stress response, response to external stimuli, response to endogenous stimuli, response to abiotic stimuli, regulation of molecular function, regulation of biological processes, immune response, immunological effector process, cellular response to stimuli, cell development, cell population proliferation, cell motility, cell growth, cell death, cell communication, cell adhesion, catabolic processes, biosynthetic processes, and development of anatomical structures (Figure 6D). These results indicate a metabolic and cellular response in growth and the immune system in the digestive gland of *M. chilensis* under hypoxic conditions. In comparison, reoxygenation showed fewer annotated GO terms relative to hypoxia. However, transcript counts per term were similar (Figure 6D). Terms identified include response to external stimuli, protein folding, molting cycle, immune response, and cell adhesion. In addition, terms that were not found in hypoxia were recorded in reoxygenation, such as glycosylation, embryonic implantation, digestion, demethylation, and coagulation. These findings revealed a marked difference in the metabolic and immune system response of the digestive gland of *M. chilensis* under hypoxic conditions compared to reoxygenation.

### 3.6. Differential Expression ANALYSIS of transcripts Observed in the Adductor Muscle of M. chilensis under Hypoxic and Reoxygenation Conditions

**Figure 7:**
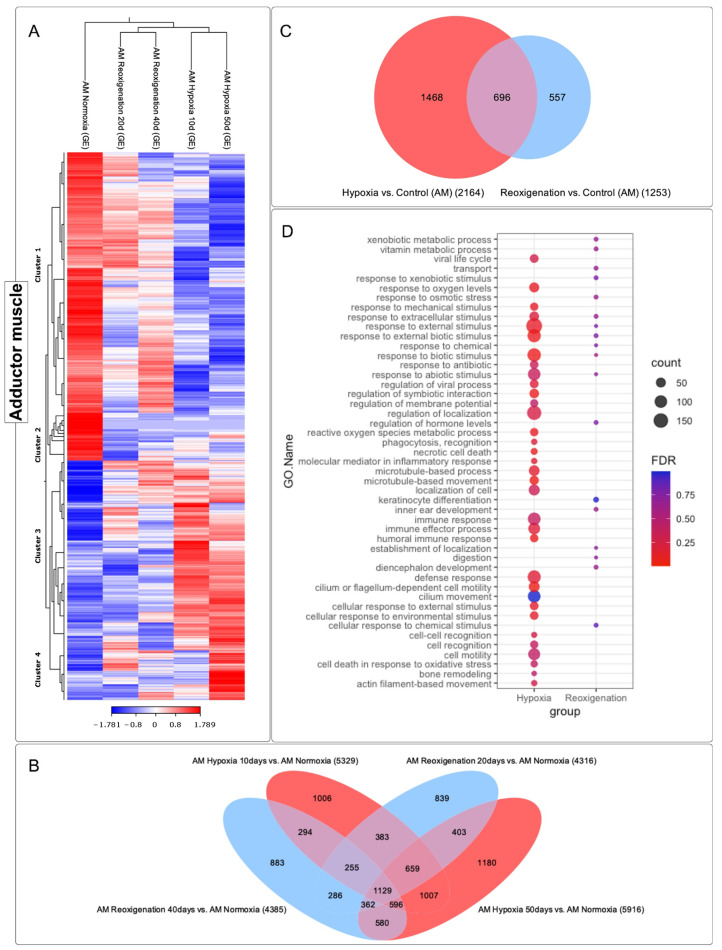
Gene expression analysis of the differentially expressed genes during hypoxia in the adductor muscle of *M. chilensis*. (**A**) Heatmap and hierarchical clustering show the most robust upregulated genes in red and downregulated genes in blue. The dendrogram clusters genes with similar expression change patterns. (**B**) Venn diagram shows the number of unique and overlapping transcripts differentially expressed after exposure to hypoxia–reoxygenation. Sampling was performed at 10 and 50 days of hypoxia and 20 days and 40 days of reoxygenation. A total of 1180 transcripts were differentially expressed in the adductor muscle at 50 days subjected to hypoxia and were not differentially expressed in any other sampling. There were 1129 most common overlapping transcripts, which were differentially expressed at both 10 and 50 days under hypoxia conditions, as well as at 20 and 40 days under reoxygenation conditions. Genes regulated in hypoxia are highlighted in red, while those regulated in reoxygenation are highlighted in blue. (**C**) Venn diagram shows overlapping genes differentially expressed compared with aerated controls in hypoxia and reoxygenation in adductor muscle. Genes regulated in hypoxia are highlighted in red, while those regulated in reoxygenation are highlighted in blue. (**D**) Function annotation and Gene Ontology (GO) term enrichment analysis of DEGs in upregulated and downregulated genes in response to hypoxia. FDR: false discovery rate. The most representative and significant molecular functions, biological processes, and cellular components are represented. The circumference size indicates the number of DEGs associated with the process, and the dot color indicates the significance of the enrichment (FDR-corrected *p*-values).

The adductor muscle exhibited significant changes in its gene expression profile after 10 and 50 days of hypoxia, which was reflected in the heatmap (Figure 7A). However, during days 20 and 40 of reoxygenation, modifications in the transcriptome were less marked compared to what was observed during hypoxia (Figure 7A). During the reoxygenation phase, a recovery and adaptation process was recorded. Four groups of transcripts with different expression patterns could be identified in the adductor muscle (Figure 7A). Specifically, clusters 3 and 4 consisted of upregulated transcripts, while clusters 1 and 2 included downregulated transcripts (Figure 7A). A total of 9862 transcripts were identified that underwent regulation during both the 10- and 50-day hypoxia periods and the 20- and 40-day reoxygenation periods, and a core group of 1129 transcripts was found that remained consistent across all periods of hypoxia and reoxygenation (Figure 7B). The highest number of differentially expressed transcripts was observed at 50 days of hypoxia, followed by hypoxia at 10 days, reoxygenation at 40 days, and finally reoxygenation at 20 days (Figure 7B). These results suggested a more pronounced response of the transcriptome to hypoxia compared to reoxygenation and also indicated a greater sensitivity of this tissue to hypoxic and reoxygenating conditions over time (Figure 7B). Venn diagram analysis (Figure 7C) highlighted that there was a more significant number of differentially expressed transcripts in the hypoxic conditions compared to the reoxygenation conditions. In the adductor muscle, a more substantial number of transcripts were observed that were only expressed during hypoxia, in contrast to those that showed exclusive regulation in reoxygenation (Figure 7C). Interestingly, transcripts that experienced upregulation in both the hypoxia and reoxygenation periods were found to be more numerous compared to those that were exclusively manifested during reoxygenation.

### 3.7. GO Enrichment Analysis in the Adductor Muscle of M. chilensis under Hypoxic and Reoxygenation Conditions

To identify the altered biological processes in the adductor muscle of *M. chilensis* under hypoxic and reoxygenating conditions, GO enrichment analysis was performed (Figure 7D). This analysis revealed that hypoxia is significantly associated with a variety of biological processes, including response to oxygen, response to extracellular stimuli, response to external stimuli, response to abiotic stimuli, regulation of membrane potential, regulation of localization, recognition of phagocytosis, immune response, effector process of the immune response, humoral immune response, defense response, cellular response to external stimuli, cellular response to environmental stimuli, cellular recognition, cellular mobilization, cell death in response to oxidative stress, bone remodeling, and movement based on actin filaments (Figure 7D). In comparison, reoxygenation showed a lower number of annotated GO terms relative to hypoxia (Figure 7D). Furthermore, the transcript count at the terms was lower (Figure 7D). Common terms identified in reoxygenation and hypoxia included response to extracellular stimuli, response to external stimuli, response to abiotic stimuli, and hormone level regulation (Figure 7D). In addition, terms that were not found in hypoxia were recorded in reoxygenation, such as vitamin metabolism, transport, regulation of hormone levels, keratinocyte differentiation, localization establishment, and digestion. These results suggest that hypoxia induced a series of biological changes in the adductor muscle of *M. chilensis*. These changes included activating the immune response, the cellular response to external stimuli, and regulating energy metabolism. Reoxygenation, however, was associated with less activation of these processes.

### 3.8. Identification and Expression of the mTOR Signaling Pathway in M. chilensis under Hypoxia

**Figure 8:**
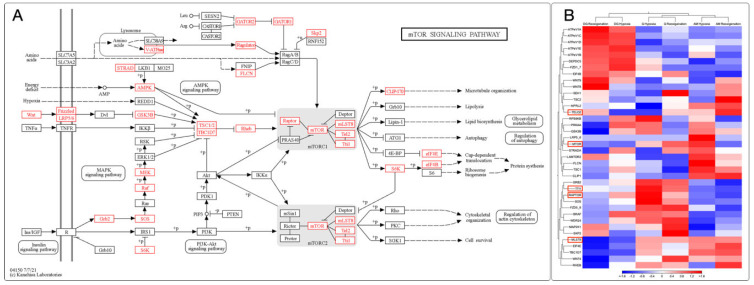
Illustration of the mTOR signaling pathway in *M. chilensis* under hypoxia conditions. (**A**) Interaction between hypoxia and the mTOR signaling pathway in gills. (**B**) Heatmap and hierarchical clustering to show MTOR under hypoxic conditions and reoxygenation in the digestive gland (DG), gill (G), and adductor muscle (AM).

In this study, gene expression analysis was carried out in the gills, digestive gland, and adductor muscle of *M. chilensis* under hypoxic conditions. Through the transcripts generated from sequencing, 38 key genes involved in different stages of the mTOR signaling pathway were identified in *M chilensis* under hypoxia. These findings allowed the construction of a putative model of the mTOR signaling pathway under hypoxic conditions (Figure 8A). Some key genes stood out in these clusters. Different regulatory patterns were recorded in several tissues in response to hypoxia and reoxygenation (Figure 8B). For example, TELO2 showed similar behavior in the digestive gland and gills, being downregulated in hypoxia and upregulated in reoxygenation. In contrast, in the adductor muscle, it was upregulated in both conditions. The MTOR gene showed differential regulation in the three tissues, with both downward and upward responses depending on hypoxia and reoxygenation.

### 3.9. Transcriptional Response of HIF and PHD in Different Tissues of M. chilensis during Hypoxia and Reoxygenation Phases

**Figure 9:**
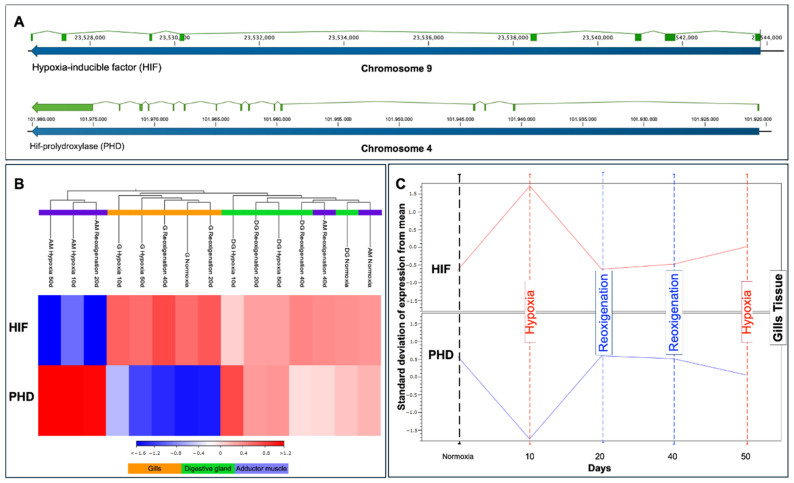
Comparative analysis of HIF and PHD genes in *M. chilensis* under hypoxia conditions. (**A**) Localization of Hif-α and Phd on chromosomes. HIF is located in chromosome 9, and PHD in chromosome 4. (**B**) Heatmap and hierarchical clustering to show HIF and PHD mRNA regulation patterns in gills, digestive gland, and adductor muscle under hypoxia and reoxygenation conditions. (**C**) Standard deviation of HIF and PHD expression in gills versus mean expression in reoxygenation and hypoxia. At 10 days in hypoxia, HIF is upregulated, and PHD is downregulated. The red line represents HIF, while the blue line represents PHD.

The HIF gene is located on chromosome 9, while PHD resides on chromosome 4 (Figure 9A). The relative expression levels of HIF and PHD mRNA in each tissue revealed heterogeneous regulation patterns (Figure 9B). In the gill, HIF experienced upregulation in all treatments including the control group, while PHD showed downregulation in all treatments including the control group (Figure 9B). An increase in the expression of transcripts for HIF and a decrease in transcripts for PHD was observed in the gill after the first 10 days of exposure to hypoxia, followed by a stabilization of the levels of both transcripts from day 20 to day 50 (Figure 9C). Similarly, upregulation of HIF and PHD was detected in this tissue during hypoxia compared to reoxygenation (Figure 9C). Subsequently, after reoxygenation on day 40, the upregulation of HIF and PHD was higher compared to reoxygenation on day 20 (Figure 9C). In the adductor muscle, an upregulation of HIF and PHD was observed in the control group and in reoxygenation at 40 days, while in the rest of the treatments, HIF was downregulated and PHD was upregulated (Figure 8B). HIF and PHD exhibited upregulation in the digestive gland in all cases (Figure 9B).

### 3.10. Identifying and Expressing Transcripts in the Toll-like Receptor, Citrate Cycle (TCA), and Apoptosis Signaling Pathways in the Gills of M. chilensis under Hypoxia

**Figure 10:**
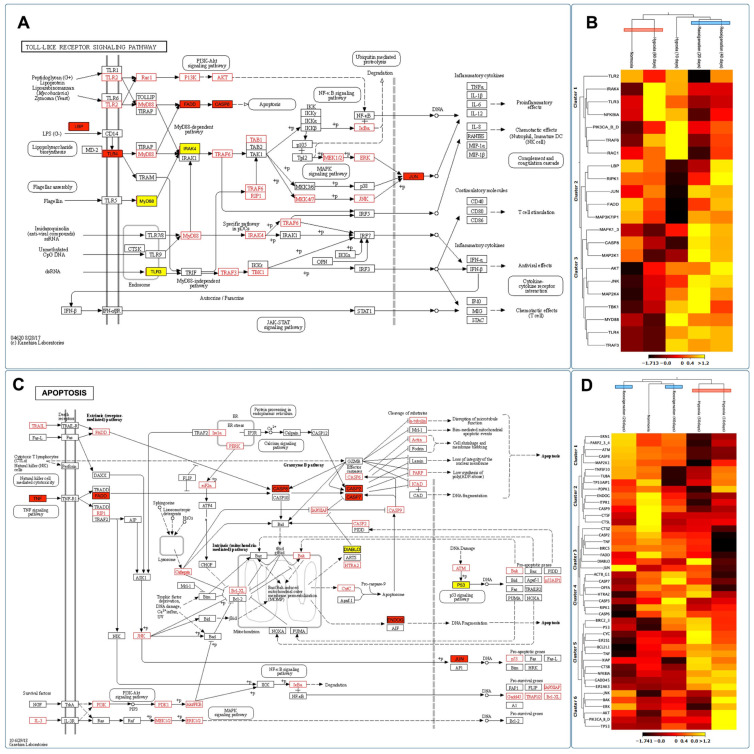
Illustration of the Toll-like receptor signaling and apoptosis pathways in *M. chilensis* gills under hypoxia conditions. (**A**) Toll-like receptor is an inducible transcription factor that inactivates JUN, thereby regulating the hypoxia process. (**B**) Heatmap and hierarchical clustering show JUN downregulated under hypoxic conditions and upregulated under reoxygenation. (**C**) In the intrinsic apoptotic pathway, cellular stress leads to Bak oligomerization, which permeates the mitochondrial outer membrane, releasing apoptogenic factors, including cytochrome c. In the cytosol, cytochrome c activates caspase 9, which cleaves and activates executioner caspases, such as caspase 6 and 7. In the extrinsic apoptotic pathway, ligating death receptors lead to the recruitment of adaptor proteins and subsequent activation of caspase 8, which activates executioner caspases. In addition, activation of apoptosis by the extrinsic pathway was mediated by TNF-α. (**D**) Heatmap and hierarchical clustering show genes regulated under hypoxia conditions and reoxygenation in the apoptosis pathway.

**Figure 11:**
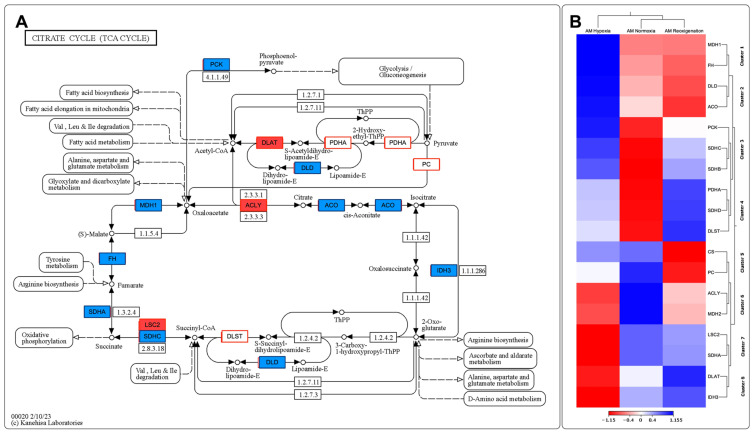
Illustration of the citrate cycle (TCA cycle) in *M. chilensis* gills under hypoxia conditions (**A**). Under hypoxic conditions, the metabolic activity shifted from oxidative to glycolytic metabolism. This metabolic switch was primarily regulated by increased hypoxia-inducible factor (HIF) activity and enhanced glycolysis. Genes upregulated of the cycle are highlighted in blue. Genes downregulated of the cycle are highlighted in red. (**B**) Heatmap and hierarchical clustering show genes related to the citrate cycle under hypoxic and reoxygenation conditions. Genes with similar expression patterns are grouped through hierarchical clustering, providing insights into the coordinated regulation of genes involved in the citrate cycle under hypoxia and reoxygenation conditions.

Through the de novo assembly of the transcripts obtained by sequencing, key genes involved in various stages of the Toll-like receptor signaling pathway, the citrate cycle (TCA), and apoptosis in the context of hypoxia were identified, which allowed the construction of putative models for each of these pathways (Figure 10A,C and Figure 11A). The results revealed significant changes in the expression of genes related to Toll-like receptor signaling pathways, citrate cycle (TCA), and apoptosis in the gills of *M. chilensis* under hypoxia. In the case of the Toll-like receptor signaling pathway, three hierarchical clusters of RPKM values were generated, allowing a detailed view of its regulation to be obtained (Figure 10B). This analysis highlighted significant changes in gene expression of genes related to this pathway. Cluster 1, which includes genes such as TLR2, NF-κB, and RAC1 related to immune response, showed downregulation during the reoxygenation process at 20 days (Figure 10B). Regarding the apoptosis signaling pathway, six clusters were generated to identify differentially expressed transcripts (Figure 10D). Cluster 2 of this pathway was composed of the genes ENDOG, ITPR 1, CASP 9, CTSF, and CTSL, which play a critical role in cell death (Figure 10C). These genes exhibited upregulation under normoxia conditions and downregulation under hypoxia and during reoxygenation (Figure 10D). Finally, regarding the citrate cycle (TCA), eight hierarchical clusters were generated to identify differentially expressed transcripts (Figure 11B). This also revealed significant changes in the gene expression of genes related to this pathway. Cluster 1 of this pathway was composed of the MDH1 and FH genes, and cluster 2, formed by the DLD and ACO genes, plays a fundamental role in the enzymatic reactions that convert carbohydrates, fats, and proteins into energy. These genes showed upregulation under hypoxic conditions and downregulation during normoxia and reoxygenation (Figure 11B).

## 4. Discussion

Oxygen is a dominant ecological factor affecting benthic organisms’ biomass and species composition [98]. Therefore, the effect of hypoxia can be dramatic and have essential consequences on benthic species that are not adapted to low dissolved oxygen environments for extended periods [22,99]. The Chilean mussel’s capacity for adaptation to hypoxia has yet to be well known. Thus, this study aimed to analyze the transcriptome of *M. chilensis* and elucidate the specific gene expression in three tissues (gills, digestive gland, and adductor muscle) subjected to hypoxia. Most Chilean aquaculture farms are in Chiloé Island in the Los Lagos Region. Therefore, the mussels were collected from a farm in Puerto Montt, where there was a risk of hypoxic events caused by upwelling and eutrophication. This study is the first conducted on the effect of hypoxia in *M. chilensis*. 

Hypoxia activates various molecular pathways in bivalve mollusks as an adaptive mechanism to restore oxygen homeostasis [100]. In recent decades, transcriptomic responses to hypoxia have been studied in several marine bivalve species [82,101,102,103]. In this study, a transcriptomic reaction was observed in the gills, adductor muscle, and digestive gland in response to hypoxic stress, indicating the importance of these tissues in regulating hypoxia in the Chilean mussel. Different tissue-specific changes in gene expression were observed in the three analyzed tissues, suggesting a tissue-specific response in the mussel. The insulin-like growth factor binding protein complex acid labile subunit gene was expected to be expressed in all tissues. This is a growth factor known to activate signal transduction pathways that lead to the expression of HIF-1α, thereby stabilizing HIF-1α under normoxic conditions [104].

According to the PCA of differential expression, PC1 and PC2 play a significant role in explaining the global variation in gene expression. The findings also reveal noticeable disparities in expression patterns across different tissue types, providing valuable insights into the underlying gene expression patterns in the studied tissues. These results help identify crucial components responsible for gene expression variation and highlight tissue-specific differences in the transcriptome, consistent with previous studies [3,29].

For aerobic organisms, post-hypoxic reoxygenation is associated with additional challenges due to the energy needed to restore cellular homeostasis and replenish energy stores [29]. The re-establishment of oxygen and nutrient supply, along with the restart of mitochondrial energy production, leads to oxidative damage through an increase in reactive oxygen species (ROS) from the mitochondrial electron transport system (ETS) [29,105]. However, a partial recovery of the gene transcription profiling after hypoxia was observed during reoxygenation, consistent with prior research [29,106]. This is the first time it has been reported that hypoxia generates a more significant response to regulatory patterns than reoxygenation.

In contrast to other studies where changes are more pronounced during reoxygenation, such as in the profile of apoptotic, inflammatory, and autophagic biomarkers [29], the current findings indicate that physiological and cellular stress associated with reoxygenation typically occurs within minutes to hours after the return of oxygen [3]. These findings highlight the importance of regulating cell survival pathways in tolerating intermittent hypoxia in marine bivalves and demonstrate the effectiveness of molecular markers in sentinel marine bivalves for monitoring hypoxia-induced stress in estuarine and coastal habitats.

This section addresses the increasing contribution of the unfolded protein response (UPR) in the endoplasmic reticulum (ER) under hypoxic conditions for the first time in bivalve mollusks. The ER is a dynamic intracellular organelle with multiple critical functions in a wide range of processes, including cellular homeostasis; development; the stress response; protein synthesis, folding, modification, and transport; lipid transport; storage of calcium ions within its lumen and their regulated release to the cytoplasm; metabolic regulation; reactive oxygen species (ROS) signaling; autophagy; and signaling and adaptation to constantly changing environments [107]. High protein synthesis, folding, modification, and transport levels are required to initiate and maintain effective immune responses, all coordinated by the endoplasmic reticulum [108]. It is important to note that various conditions inside and outside the cell can affect the ability of this organelle to process proteins, resulting in a state known as “endoplasmic reticulum stress”, which activates the unfolded protein response (UPR) [108]. The UPR is a cellular signaling system that readjusts the folding capacity of the ER to restore protein homeostasis in response to endoplasmic reticulum stress [109]. Unfolded or misfolded proteins activate the UPR pathway to cope with ER stress, activating a series of cell death pathways [110]. Apoptotic proteins such as caspase 3, calpains, and cytochrome c interact with and regulate IP3Rs, playing a crucial role in apoptotic cell death [111]. On the other hand, increased ROS levels result in misfolded/unfolded proteins accumulating, activating the unfolded protein response (UPR) [112]. Furthermore, abnormal activation of the UPR may contribute to the development of various diseases, such as neoplasia and metabolic disorders [108].

Endoplasmic reticulum (ER) stress and the activation of the unfolded protein response (UPR) have been associated with intracellular lipid accumulation [113]. The ER, as a site of synthesis of a variety of essential lipids, including cholesterol, triacylglycerols, and phospholipids, plays a critical role in the lipid homeostasis of organisms, including bivalve mollusks [114,115].

Furthermore, the fact that the proteins and lipids that make up the Golgi apparatus originate in the endoplasmic reticulum (ER) underlies this organelle’s importance in synthesizing and processing molecules destined for cellular secretion [116]. The close association between the UPR and lipid homeostasis in the context of metabolic diseases suggests the possible involvement of these processes in the pathogenesis of various diseases [105,117]. Despite significant advances in treating some pathologies, there is still a gap in our complete understanding of the role of the UPR. Therefore, additional research is required to explore the broad therapeutic opportunities that UPR could offer to treat several diseases [118].

In the GO enrichment analysis, differentially expressed transcripts assigned to KEGG pathways related to metabolism, cellular processes, and environmental sensing were observed, in addition to ubiquitin binding as shown in Figure 3C, consistent with what was found in other studies [119]. 

A differential expression of transcripts associated with enzymes involved in the metabolism of amino acids, such as V-ATPase as shown in Figure 8A and in the amino acids alanine, aspartate, glutamate, tyrosine, and arginine as shown in Figure 11A, was also observed. Amino acids are essential in the anaerobic metabolism of bivalves [119]. Protein catabolism has also been demonstrated in bivalves during hypoxia as a potential mechanism for maintaining amino acid stores, as amino acids are essential in the osmotic balance [3,119,120]. In the present study, differential expression of transcripts associated with amino acid metabolism was observed; for example, MDH1 was upregulated in hypoxia and downregulated in normoxia and reoxygenation, PCK was upregulated in hypoxia and downregulated in reoxygenation, ACLY was downregulated in hypoxia and reoxygenation and upregulated in normoxia, and IDH3 was downregulated in hypoxia and upregulated in reoxygenation and normoxia. This mechanism may be associated with maintaining free amino acids as an essential strategy for survival under hypoxic conditions [119]. Aspartate is usually depleted during anaerobic transamination reactions [121].

Figure 11 records an underexpression of transcripts associated with critical enzymes related to aerobic respiration and the progression of the citric acid cycle, which is consistent with what was found in other studies [119].

The increased upregulation of ACO during hypoxia, concomitant with a decrease in normoxia and reoxygenation, points to a critical role of this enzyme in isocitrate production under conditions of low oxygen availability. Similarly, the upregulation of PCK during hypoxia and a decrease in normoxia and reoxygenation indicate its involvement in pyruvate production under hypoxic conditions. These changes in transcript expression are significant findings, given that these genes are associated with aerobic respiration, and their modification in expression could indicate an alteration in aerobic metabolic pathways [119].

Furthermore, it is essential to highlight that the UPR, an adaptive response to hypoxia, nutrient deprivation, and stress, plays a crucial role in several types of neoplasia, acting as a dynamic promoter in developing these diseases. This finding suggests that regulation of the UPR could be a promising strategy for cancer treatment [118].

A fundamental characteristic of neoplastic cells is their ability to metabolize glucose rapidly and their high proliferation rate [122]. This phenomenon may result in poor vascularization of the tumor mass, leading to insufficient oxygen supply [122]. Furthermore, neoplastic cells require high levels of protein synthesis to maintain their uncontrolled growth and proliferation [123]. The endoplasmic reticulum (ER) stress and activation of the UPR typically trigger these oncogenic conditions [124].

Several oncogenic, transcriptional, and metabolic abnormalities in various malignancies collaborate to create hostile microenvironments that perturb ER homeostasis in malignant cells [124]. These changes induce a persistent stress state in the ER, which has been shown to regulate multiple tumor-promoting characteristics in neoplastic cells [124]. Therefore, ER stress sensors’ abnormal activation and subsequent signaling pathways have emerged as critical tumor growth and metastasis regulators [124]. Physiological or pathological activation of the UPR can affect the survival, metabolism, function, and fate of immune cells [108].

Efficient cellular function depends on oxygen availability to maintain normal cell function. However, using oxygen at this level generates free radicals, which can lead to oxidative stress. An intricate network of surveillance mechanisms is required to regulate this system effectively to maintain adequate oxygen homeostasis [125]. Maintaining the organism’s homeostasis depends on integrating external and systemic signals and the ability to perceive cellular perturbations to trigger adaptive responses [117].

Furthermore, enriched with a high concentration of mitochondria, cells exhibit a demanding requirement for glucose as an energy substrate. This heightened energy demand generates intense mitochondrial activity, inevitably producing free radicals as a byproduct. Cells resort to adaptive stress response pathways, which allow them to survive oxidative stress and minimize cellular damage to preserve this balance. These stress response pathways depend on optimal endoplasmic reticulum (ER) function and activation. The UPR is a critical cellular pathway that maintains normal ER function and cell survival [125]. The UPR transmits information about the folding state of proteins to the nucleus and cytosol to adjust the protein folding capacity of the cell [117].

Interestingly, the UPR consists of two opposing signaling pathways: one that promotes cell survival by reducing ER damage during stressful situations and another that induces apoptosis if the stress is prolonged or not adequately mitigated [125]. As described in Figure 3A, the homeostasis of the endoplasmic reticulum (ER) is achieved thanks to the presence of the response to misfolded proteins (UPR), which is essential for the maintenance of the integrity and function of the ER in the context of hypoxic situations. Figure 8A describes, for the first time in bivalve mollusks, the effect of the UPR on cellular metabolism by attenuating general protein translation through the phosphorylation of eIF4B activated by S6K. This downregulation reduces protein loading in the ER and increases ATP availability for processes such as protein folding and degradation, consistent with other studies [105].

Apoptosis, autophagy, translation, energy metabolism, and inflammation are fundamental cellular processes coordinated by intracellular signaling pathways, particularly the regulatory complex known as mTOR and the endoplasmic reticulum stress response (UPR) [126]. mTOR, a protein kinase, is crucial in regulating cell proliferation, survival, metabolism, and immune response. On the other hand, adenosine monophosphate-activated protein kinase (AMPK) acts as a critical sensor of cellular energy, influencing lipid homeostasis and glucose metabolism. These pathways converge in autophagy [127]. The induction of the UPR arises in response to the decrease in cellular ATP resulting from glucose deprivation, which affects the function of the endoplasmic reticulum calcium pump and intracellular calcium levels. Under prolonged endoplasmic reticulum stress, mTORC1 participates in apoptotic signaling by inhibiting the survival kinase Akt and selectively activating the JNK protein kinase pathway [126].

When the ability of the UPR to maintain proteostasis is overwhelmed due to ER stress, it triggers activation of the canonical apoptosis pathway, which involves conformational activation of proapoptotic members of the BCL-2 family in the mitochondria, BAX, and BAK, with simultaneous assembly of the apoptosome and activation of executioner caspase 3. The BCL-2 family proteins, including PUMA and NOXA, are essential factors mediating ER-stress-induced apoptosis in various cellular systems, where activation mechanisms involve only transcriptional regulation and post-translational modifications of proapoptotic BCL-2 proteins. The UPR is widely involved in the signal transduction of inflammatory responses. PERK-mediated phosphorylation of eIF2α attenuates global protein synthesis and promotes the activation of nuclear factor-κB to induce pro-inflammatory genes [117]. PERK-mediated phosphorylation of eIF2α is observed in Figure 10C, in addition to the activation of the nuclear factor-κB signaling pathway.

In genomics and molecular biology, regulating gene expression in response to oxygen availability is vital for cellular adaptation to changing conditions caused by climate change. In this context, hypoxia-inducible factor-1 (HIF-1) emerges as a central figure in orchestrating cellular responses to hypoxia as an adaptive response. Under normoxia conditions, HIF-1α, one of the subunits of HIF-1, is constantly synthesized but undergoes rapid degradation mediated by the HIF–prolyl hydroxylase (PHD) complex. This process depends on intracellular oxygen since PHD requires molecular oxygen as a cofactor. Hydroxylation of specific residues on HIF-1α by PHD marks the protein for proteasomal degradation. Therefore, HIF-1α is maintained at low levels under normoxia conditions due to its continuous degradation, which prevents the activation of target genes associated with the hypoxia response [128].

However, in a hypoxic environment, oxygen availability decreases, inhibiting PHD activity. Lack of oxygen prevents the hydroxylation of HIF-1α, stabilizing this subunit. Stabilized HIF-1α undergoes translocation to the cell nucleus, where it forms an active complex with HIF-1β, and together, they act as a transcription factor that binds to hypoxia response regulatory elements (HREs) in target gene promoters. The activation of HIF-1 triggers a cascade of molecular events that impact cellular physiology in multiple ways. HIF-1-regulated target genes are involved in anaerobic glycolysis, cell signaling, and oxidative phosphorylation. This allows the cell to adapt to hypoxia by increasing glucose uptake and utilization, improving cell survival, and modulating the immune response. These results, in addition to suggesting post-translational regulation of HIF-α through PHD, strongly indicate that an oxygen-dependent mechanism plays a fundamental role in the stability and activity of HIF-α. Although HIF-α is regulated through PHD, the latter is controlled by HIF-α, forming a negative feedback loop. The results of the present study, as described in Figure 6B, show a differential transcriptional response of Hif-α and PHD in the tissues analyzed. Gills showed a prominent expression that was less marked in the digestive gland, which is consistent with other studies. The high sensitivity of the oxygen-sensing pathway in the gills to hypoxia can be attributed to their direct exposure to seawater, thus making them the first tissue to feel the detrimental effects of hypoxia [82]. 

Furthermore, these structures play a fundamental role in regulating essential biological processes, such as gas transfer and osmotic balance, and activating adaptive responses to hypoxia [128]. The upregulation of Hif-α in the gills indicates an adaptation to hypoxic conditions. At the same time, the decrease in PHD in this tissue could be involved in stabilizing Hif-α.

In the adductor muscle, after 40 days of reoxygenation, transcript expression resembles the control group, suggesting long-term adaptation. In the subsequent reoxygenation phase at 20 days (Figure 6C), the expression of hif-α mRNA was not affected by normoxia, while the reduction observed in the amount of HIF-α protein at 40 days of reoxygenation could be attributable to degradation by PHD activity, being consistent with other studies [128]. Furthermore, the significant role played by HIF-α may be restricted to initiating the sequence of events that occur a few hours after oxygen deprivation [128]. There were no significant differences in the digestive gland regulation of Hif-α and PHD in the different treatments.

In summary, this study’s findings revealed that the gills and adductor muscles were more sensitive to the effects of hypoxia than the digestive gland. These results provided a better understanding of the regulation of Hif-α and PHD in various tissues. They established a basis for future investigations into the function of these genes in adaptive and pathological responses.

Marine organisms subjected to hypoxia face the critical challenge of reduced energy supply due to oxygen deficiency, as energy is essential for the normal functioning of all biological systems [129]. To cope with energy shortages under hypoxic conditions, these organisms rely heavily on hypoxia-inducible factor-1 (HIF-1), which plays a crucial role in regulating oxygen transport genes and energy production through processes such as glycolysis [130]. Various investigations in mussels have reported findings on the impact of hypoxia on gene expression related to oxidative stress and the activity of antioxidant enzymes [17,131]. This is directly related to increased production of reactive oxygen species (ROS) in cells, posing a potential risk of oxidative damage. Exposure to hypoxia may also have repercussions beyond cellular biochemistry [84]. In bivalve mollusks, hypoxia can inhibit gonadal development [132]. These effects can be attributed to changes in energy balance due to hypoxia, which, in turn, negatively affects reproduction and population dynamics [133]. HIF-1 activation and accumulation depend on the presence of reactive oxygen species (ROS), posing an exciting paradox [78]. Although these species are necessary for HIF-1 signaling, they can also induce oxidative stress in the organism [79]. It is in this context that superoxide dismutase (SOD) emerges as an irreplaceable enzyme. SOD specifically catalyzes the decomposition of excess superoxide, playing an essential role in protecting the organism against oxidative stress [78]. Previous research in marine animals has confirmed the importance of SOD by significantly increasing its activity under hypoxic conditions [83].

Blue mussels lack adaptive immunity, relying instead on innate immunity for survival and defense against biological and environmental threats [78]. Hypoxia negatively impacts the immunity of blue mussels by suppressing their immunocompetence [78,134]. Therefore, it is crucial to understand the ability of mussels to maintain their innate immunity under hypoxic conditions, which determines their adaptation and survival in changing and challenging environments [78]. This study is the first to record hypoxia’s effect on the mussel immune system in multiple tissues, including the gill, adductor muscle, and digestive gland. The results indicate that this effect was maintained mainly in the gill and digestive gland during reoxygenation. Interestingly, in all tissues analyzed, the impact on the immune system was more pronounced during hypoxia than reoxygenation. This finding raises important questions about adapting the mussels’ immune system to fluctuations in oxygen levels. The specificity of the immune response in the gill and digestive gland suggests the existence of unique regulatory mechanisms in these tissues, which could be related to their direct exposure to variations in oxygen concentration. It is crucial to highlight that, uniformly in all tissues analyzed, hypoxia caused a more pronounced impact on the immune system than reoxygenation. This fact highlights the importance of thoroughly understanding the effects of hypoxia on mussel immunity and its potential implications for the health of marine populations in the context of environmental change. This study lays the foundation for future research to unravel the molecular mechanisms underlying these specific immune responses in mussels, which could have significant implications for the conservation and management of marine ecosystems.

The blue mussel is known for its outstanding tolerance to hypoxia [78]. It is considered a conformist organism in terms of its response to dissolved oxygen in the environment [78]. This behavior means it adapts to the amount of oxygen available in its environment without adequate regulation, and its respiration rate varies directly with the external oxygen level [78]. When the dissolved oxygen concentration drops below 5–6 mg L-1, the blue mussel decreases its respiration rate and reduces its total energy requirement [78]. This ability to adjust their metabolism in the face of hypoxic conditions is an impressive example of an adaptation of marine organisms to changing and challenging environments [78]. In previous research on the gills of *Mytilus galloprovincialis*, it has been suggested that these mollusks have an adaptive response to hypoxic conditions [79]. In the present study, a notable decrease in gene expression in the gills was observed when comparing the first and third exposures of mussels to hypoxia, as evidenced in Figure 2. This finding reinforces the growing body of evidence showing that mytilids can adapt to environments with reduced oxygen levels, hinting at a genomic response to these challenging conditions. It is essential to highlight that the adaptation mechanism differs between different tissues since the digestive gland and the adductor muscle do not follow this same trend observed in the gills. This suggests that the gill, being the tissue most exposed to hypoxia due to its function in gas exchange, is the one that adapts most quickly to these adverse conditions. These findings provide valuable insight into the molecular mechanisms underlying mussel adaptation to hypoxia and highlight the importance of understanding how different tissues can respond differently to the same environmental challenges.

## 5. Conclusions

For the first time, this study investigated the transcriptomic response of three tissues in *M. chilensis* under hypoxic stress using the Illumina platform technology. It provides new insights and a comprehensive understanding of the molecular mechanisms underlying tolerance and resistance to hypoxia in the Chilean mussel. The differential expression of transcripts detected in the gills, digestive gland, and adductor muscle offers a list of candidate adaptive genes that control multiple fitness-related traits in populations subjected to hypoxia. It highlights the adaptation mechanisms of the Chilean mussel to hypoxia induced by climate change. These candidate adaptive genes could be used to select future breeding lines of *M. chilensis* broodstock for laboratory production of seedlings intended for cultivation or restocking.

Moreover, the identified Gene Ontology terms, such as negative regulation of endoplasmic reticulum unfolded protein response and TORC1 signaling, offer valuable insights into the molecular mechanisms underlying the adaptation of *M. chilensis* to hypoxic conditions. The negative regulation of endoplasmic reticulum unfolded protein response suggests potential mechanisms for downregulating cellular stress and maintaining protein homeostasis under oxygen-deficient conditions. The involvement of TORC1 signaling indicates a potential role in coordinating cellular responses to hypoxia, providing insights into metabolic pathway regulation and environmental adaptation. The GO enrichment analysis revealed that Wnt and β-catenin are key signaling pathways involved in the adaptation mechanism of the Chilean mussel to hypoxia, offering valuable information for further investigation of critical molecules regulating hypoxia tolerance and gaining new insights into mechanisms of resistance to stress caused by hypoxia in marine bivalves. 

Furthermore, these findings serve as biomarkers for detecting natural seed beds and farms that have experienced hypoxic events. These new genomic resources provide tools for designing a genetic selection plan for this commercially important species in aquaculture and contribute to the sustainable expansion management of an industry threatened by climate change. Additionally, they lay the groundwork for future research using RNA-seq and gene expression patterns on proteins involved in hypoxia, which can help explain the amount of genetic information inherited through epigenetic changes and the response of mussels to other environmental stressors or pathogenic agents.

## Data Availability

The original contributions presented in the study are included in the article, further inquiries can be directed to the corresponding author.
